# Risk factors associated with attendance at postpartum blood pressure follow-up visit in discharged patients with hypertensive disorders of pregnancy

**DOI:** 10.1186/s12884-023-05780-6

**Published:** 2023-06-30

**Authors:** Jingjing Li, Qin Zhou, Yixuan Wang, Lufen Duan, Guangjuan Xu, Liping. Zhu, Liping Zhou, Lan Peng, Lian. Tang, Yanxia. Yu

**Affiliations:** 1 Department of Pharmacy, The Affiliated Suzhou Hospital of Nanjing Medical University, Suzhou Municipal Hospital, Jiangsu Suzhou, 215002 China; 2 Department of Obstetrics, The Affiliated Suzhou Hospital of Nanjing Medical University, Suzhou Municipal Hospital, Jiangsu Suzhou, 215002 China; 3 Office of Clinical Trial Institutions, The Affiliated Suzhou Hospital of Nanjing Medical University, Suzhou Municipal Hospital, Jiangsu Suzhou, 215002 China

**Keywords:** Hypertension in pregnancy, Predictors, Postpartum, Blood pressure follow-up, Attendance

## Abstract

**Background:**

This study aims to investigate the risk factors for not returning to postpartum blood pressure (BP) follow-up visit at different time points in postpartum discharged hypertensive disorders of pregnancy (HDP) patients. Likewise, females with HDP in China should have a BP evaluation continuously for at least 42 days postpartum and have BP, urine routine, and lipid and glucose screening for 3 months postpartum.

**Methods:**

This study is a prospective cohort study of postpartum discharged HDP patients. Telephone follow-up was conducted at 6 weeks and 12 weeks postpartum, the maternal demographic characteristics, details of labor and delivery, laboratory test results of patients at admission, and adherence to BP follow-up visits postpartum were collected. While logistic regression analysis was used to analyze the factors associated with not returning to postpartum BP follow-up visit at 6 weeks and 12 weeks after delivery, the receiver operating characteristic (ROC) curve was drawn to evaluate the model’s predictive value for predicting not returning to postpartum BP visit at each follow-up time point.

**Results:**

In this study, 272 females met the inclusion criteria. 66 (24.26%) and 137 (50.37%) patients did not return for postpartum BP visit at 6 and 12 weeks after delivery. A multivariate logistic regression analysis identified education level of high school or below (OR = 3.71; 95% CI = 2.01–6.85; *p* = 0.000), maximum diastolic BP during pregnancy (OR = 0.97; 95% CI = 0.94–0.99; *p* = 0.0230)and delivery gestational age (OR = 1.12; 95% CI = 1.005–1.244; *p* = 0.040)as independent risk factors in predicting not returning to postpartum BP follow-up visit at 6 weeks postpartum, and education level of high school or below (OR = 3.20; 95% CI = 1.805–5.67; *p* = 0.000), maximum diastolic BP during pregnancy (OR = 0.95; 95% CI = 0.92–0.97; *p* = 0.000), delivery gestational age (OR = 1.13; 95% CI = 1.04–1.24; *p* = 0.006) and parity (OR = 1.63; 95% CI = 1.06–2.51; *p* = 0.026) as risk factors for not returning to postpartum BP follow-up visit at 12 weeks postpartum. The ROC curve analysis indicated that the logistic regression models had a significant predictive value for identify not returning to BP follow-up visit at 6 and 12 weeks postpartum with the area under the curve (AUC) 0.746 and 0.761, respectively.

**Conclusion:**

Attendance at postpartum BP follow-up visit declined with time for postpartum HDP patients after discharge. Education at or below high school, maximum diastolic BP during pregnancy and gestational age at delivery were the common risk factors for not returning for BP follow-up visit at 6 and 12 weeks postpartum in postpartum HDP patients.

**Supplementary Information:**

The online version contains supplementary material available at 10.1186/s12884-023-05780-6.

## Introduction

Hypertensive disorders of pregnancy (HDP) represents one of the worldwide leading causes of maternal and perinatal mortality and a major cause of postpartum morbidity, mortality, and readmission [1–7], accounting for 6.9% of maternal deaths in the United States between 2011 and 2016, and with a high associated cost burden [6, 8−9]. HDP are a group of diseases, including gestational hypertension, preeclampsia, severe preeclampsia, chronic hypertension, chronic hypertension with superimposed preeclampsia, chronic hypertension with superimposed severe preeclampsia, eclampsia, or hemolysis elevated liver enzymes and low platelets (HELLP) syndrome [1–2]. Females that develop HDP are at a 2–4 fold increased risk for chronic hypertension after the pregnancy and a doubling of the risk of cardiovascular disease later in life [10–14].

Postpartum BP monitoring and follow-up after discharge is an essential component of pregnancy care for females with HDP, as most females with HDP are discharged 72 h after delivery [7]. The American College of Obstetricians and Gynecologists (ACOG) has previously recommended that females with HDP should be monitored for BP no later than 3–10 days after delivery and comprehensive postnatal visits and transition to women’s care should be provided 4–12 weeks postpartum, timing individualized and woman-centered [15]. In China, according to Chinese guidelines for the diagnosis and treatment of hypertension and preeclampsia in pregnancy [16, 17], BP should be closely monitored within 72 h after delivery, at least 4 to 6 times a day, and postpartum women with gestational hypertension should regularly monitor their BP and monitor it for at least 42 days. Moreover, all females with HDP should measure BP, and perform other exams, including urine routine, and lipid and glucose screening 3 months postpartum, which should also be followed up for life [16, 17].

Only 52.3–63.0% of the postpartum HDP patients attended a postpartum BP visit around 6 weeks postpartum [18–19], and 24.0–49.0% attended a visit around 12 weeks postpartum [19–20]. It is therefore prudent to identify who will be less likely to monitor BP, so that interventions to increase compliance may be attempted. Then, health care providers may have a better opportunity to early identify the disease and intervene before serious consequences occur. However, to date, the extent of adherence to postpartum BP follow-up and the influence factors in postpartum discharged HDP patients in China remain unclear. The aim of this study was to identify the risk factors that associated with failure to returning for postpartum BP follow-up visit at different time points within 3 months of hospital discharge in postpartum HDP patients.

## Methods

### Study design, population, and data collection

This prospective cohort study was conducted on postpartum discharged HDP patients at the Affiliated Suzhou Hospital of Nanjing Medical University from September 2017 to December 2019. The ethics committee of the hospital approved this study (K2017037). Postpartum females with HDP were received targeted discharge education. The frequency of BP monitoring, correct home BP monitoring method, and BP goals were provided in discharge education checklist for postpartum females with HDP (see the Supplementary Appendix). Return visits were recommended at 6 and 12 weeks postpartum to monitor and record blood pressure, and for medical personnel to decide whether to adjust the antihypertensive treatment regimen, including dose reduction, discontinuation, dose increase or medication change, and to determine the need for further haematological and biochemical monitoring and management. The value of BP monitoring should be recorded in the blood pressure record book. The record should also include: the date the blood pressure was measured and the specific time the antihypertensive medication was taken (if it was being used).

The inclusion criteria of this study include (1) Patients with HDP as the discharge diagnosis. (2) Postpartum patients. (3) Patients that cooperate with telephone follow-up. The exclusion criteria were: (1) Patients who were not followed up after discharge. (2) Patients with cognitive impairment. (3) Patients with incomplete or missing clinical and laboratory information.

Telephone follow-up was conducted at 6 and 12 weeks postpartum in the discharged females with HDP. Postpartum follow-up information included if they performed a BP monitoring according to the frequency and method in the targeted discharge education, whether return postpartum BP visits were made at 6 and 12 weeks after delivery, whether BP monitored and recorded at the return visit was normal (< 140/90 mmHg), and whether antihypertensive medication was discontinued at the time of the return visit. Meanwhile, the electronic medical records of each patient were reviewed and collected, record data included maternal demographic information, hypertensive diagnosis, combined diagnosis, BP, delivery method, newborn information, and laboratory information relevant to HDP, including urine protein, platelet count (PLT), total bilirubin (TBil), serum creatinine, aspartate aminotransferase (AST), alanine aminotransferase (ALT), lactate dehydrogenase, and uric acid at admission. Then, at each follow-up time point, women with HDP who did not return for postpartum BP visit were compared to women with HDP who did return. The International Classification of Diseases (ICD-10-CM) was used for all the clinical diagnoses.

### Statistical analysis

All statistical analyses were performed using the Statistical Package for Social Sciences, version 22 (SPSS Inc. Chicago, IL, United States). The categorical variables are summarized as frequencies and proportions (%), and the Pearson’s chi-square test or the Fisher’s exact test was used to analyze categorical data. In contrast, all the continuous variables were checked for normality using the Kolmogorov-Smirnov test. While the continuous variables were summarized as the medians and interquartile ranges (IQR), the Mann-Whitney U test was used to analyze continuous data when these were not normally distributed. Continuous variables of the normal distribution are summarized as mean ± SD, and a t-test was used to analyze these continuous data. Univariate and multivariate logistic regression analyses were used to explore potential risk factors for not returning to postpartum BP visit at each follow-up time point in discharged patients with HDP, and two-tailed *P* values of < 0.05 was considered statistically significant. Variates with *P* values of < 0.1 on the univariate analyses were included in the multivariate logistic regression analysis (backward procedure, based on the p-value of the predictor removed) [21]. A multivariate logistic regression analysis was used to screen for independent risk factors. The odds ratio (OR) and 95% confidence intervals (CI) were also calculated, and the independent risk factor variables were used to establish a logistic regression model and calculate prediction probabilities. While the receiver operating characteristic (ROC) curves of independent risk factors were drawn, the area under the curve (AUC), cut-off point, Youden index, sensitivity, and specificity were used to evaluate their predictive value for predicting not returning to postpartum BP visit at each follow-up time point in discharged patients with HDP [22].

## Results

**Fig. 1:**
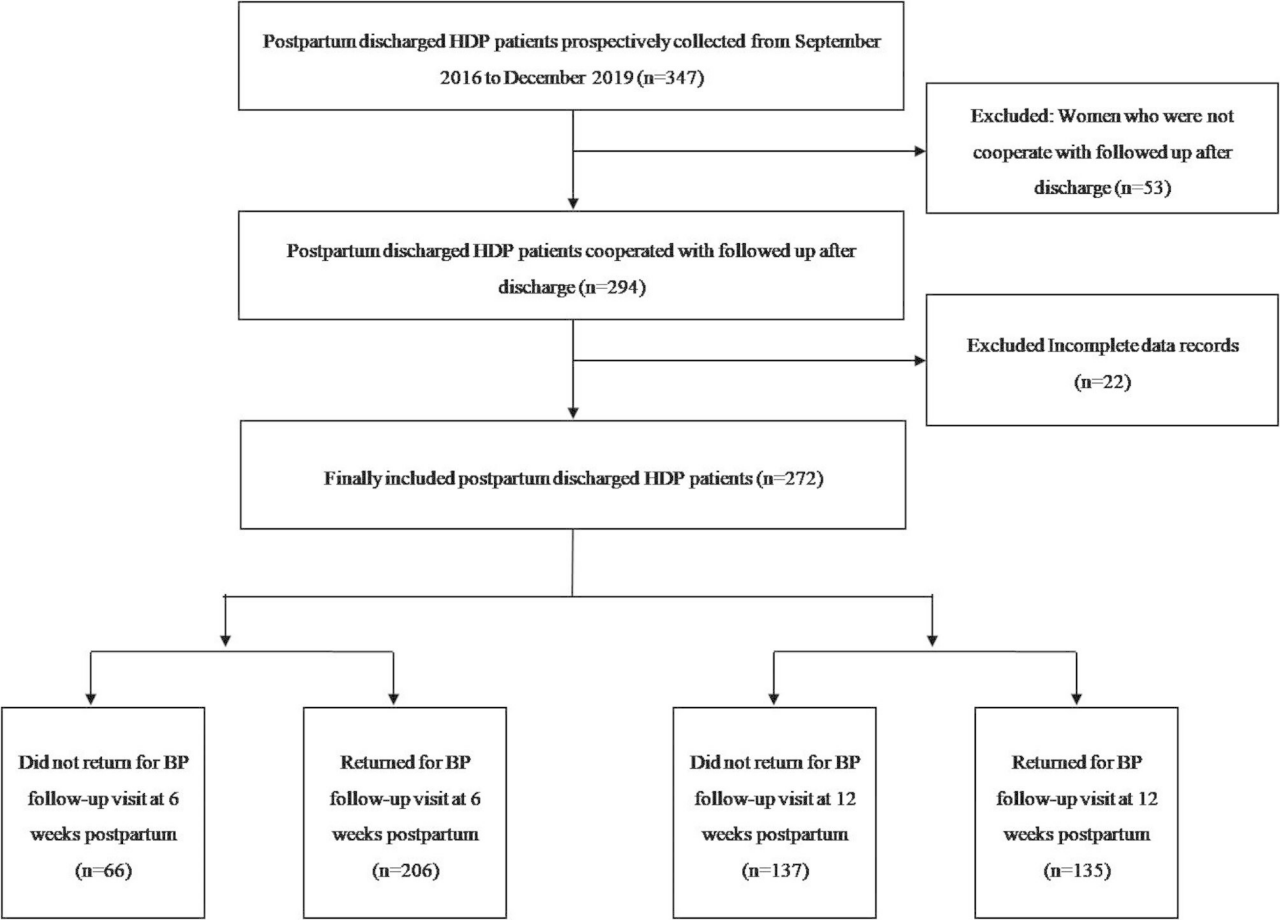
Flowcharts of patients who were included and excluded from the study

The postpartum discharged HDP patients (n = 272) were enrolled and there were 66 (24.26%) and 137 (50.37%) patients who did not return for postpartum BP visit at 6 and 12 weeks after delivery (Fig. 1). The characteristics of the study population stratified by time point of return postpartum BP visits are shown in Table 1. Women who did not return for postpartum BP visit at 6 and 12 weeks after delivery had significantly lower education levels, more parity, lower diastolic BP at admission, and lower maximum diastolic BP during pregnancy. Moreover, these patients were less likely to have been diagnosed with severe pre-eclampsia, to have given birth at a later median gestational age, and to have higher TBiL levels at admission, with statistically significant differences compared to women who did return. Also, females who did not return for BP visit at 12 weeks postpartum were more likely to be diagnosed with gestational hypertension and less likely to be combined with HELLP syndrome, whereas women who did not return at 6 weeks post-delivery were less likely to be combined with other diagnosis (*P* < 0.05) (Table 1).

**Table 1:** Characteristics of the study population stratified by time point of return postpartum BP visit

**Characteristics**	Return for BP follow-up visit at 6 weeks			Return for BP follow-up visit at 12 weeks			
	Did not (n = 66)	**Did (n = 206)**	*P*-value	Did not (n = 137)	Did (n = 135)		*P*-value
Age(years), median [IQR]	30 (29, 34)	31 (28, 35)	0.368	31 (29, 36)	31 (28, 34)		0.606
BMI on admission, median [IQR] (kg·m^− 2^)	30.30 (27.30, 33.20)	29.70 (27.30, 32.70)	0.423	30.10 (27.60, 33.00)	29.7 (27.00, 32.60)		0.569
Education level of high school or below, n (%)	36 (13.24%)	69 (25.37%)	0.002	68 (25.00%)	37 (13.60%)		0.000
Family history of hypertension, n (%)	2 (0.74%)	7 (2.57%)	1.000	5 (1.84%)	4 (1.47%)		1.000
Gravidity, median [IQR]	2 (1, 3)	2 (1, 3)	0.549	2 (1, 3)	2 (1, 3)		0.419
Parity, median [IQR]	2 (1, 2)	2 (1, 2)	0.043	2 (1, 2)	2 (1, 2)		0.000
Systolic BP on admission(mmHg), median [IQR]	145 (138, 158)	149 (139, 159)	0.163	147 (138, 158)	149 (141, 163)		0.087
Diastolic BP on admission(mmHg), median [IQR]	90 (84, 97)	96 (88, 103)	0.005	93 (83, 99)	96 (89, 108)		0.000
Maximum systolic BP during pregnancy(mmHg), median [IQR]	161 (149, 171)	161 (153, 171)	0.716	160 (150, 169)	163 (155, 175.00)		0.011
Maximum diastolic BP during pregnancy(mmHg), median [IQR]	100 (89, 108)	103 (97, 110)	0.005	100 (91, 106)	106 (100, 112)		0.000
Aspirin use during pregnancy, n (%)	3 (1.10%)	23 (8.46%)	0.111	12 (4.41%)	14 (5.15%)		0.651
Magnesium sulfate use during hospitalization, n (%)	62 (22.79%)	191 (70.22%)	0.951	124 (45.59%)	129 (47.43%)		0.103
Furosemide taken postpartum, n (%)	33 (12.13%)	96 (35.29%)	0.630	62 (22.79%)	67 (24.63%)		0.470
**Main diagnosis at discharge, n (%)**							
Gestational hypertension	9 (3.31%)	17 (6.25%)	0.195	20 (7.35%)	6 (2.21%)	0.004	
Chronic hypertension	5 (1.84%)	14 (5.14%)	0.829	8 (2.94%)	11 (4.04%)	0.455	
Eclampsia	1 (0.37%)	4 (1.47%)	1.000	1 (0.37%)	4 (1.47%)	0.212	
Preeclampsia (any)	51 (18.75%)	171 (62.87%)	0.295	108 (39.71%)	114 (41.91%)	0.232	
Severe preeclampsia	28 (10.29%)	134 (49.26%)	0.001	71 (26.10%)	91 (33.46%)	0.009	
**Combined diagnosis at discharge, n (%)**							
HELLP syndrome	2 (0.74%)	14 (5.15%)	0.406	4 (1.47%)	12 (4.41%)	0.036	
Premature rupture of membranes	5 (1.84%)	7 (2.57%)	0.150	7 (2.57%)	5 (1.84%)	0.572	
Diabetes	20 (7.35%)	53 (19.49%)	0.465	38 (13.97%)	35 (12.87%)	0.736	
Other diagnosis	4 (1.47%)	34 (12.50%)	0.033	17 (6.25%)	21 (7.72%)	0.454	
Total hospitalization days(d), median [IQR]	7 (6, 11)	7 (5, 11)	0.331	7 (5, 12)	7 (5, 11)	0.245	
In vitro fertilization, n (%)	7 (2.57%)	23 8.46(%)	0.900	16 (5.88%)	14 (5.15%)	0.731	
Singleton pregnancy, n (%)	57 (20.96%)	183 (67.28%)	0.588	119 (43.75%)	121 (44.49%)	0.479	
Cesarean delivery, n (%)	54 (19.85%)	165 (60.66%)	0.759	107 (39.34%)	112 (41.18%)	0.312	
Postpartum hemorrhage(ml), median[IQR]	300 (250, 400)	300 (300, 370)	0.566	300 (250, 400)	300 (300, 350)	0.691	
Delivery gestational age(weeks), median [IQR]	36.90 (35.00, 39.10)	35.60 (32.90, 38.10)	0.005	36.60 (34.50, 38.90)	35.30 (32.30, 38.00)	0.001	
Live birth, n (%)	66 (24.26%)	203 (74.63%)	1.000	135 (49.63%)	134 (49.26%)	1.000	
**Laboratory characteristics of the study population on admission**							
Urine protein dipstick test reading ≥ 1+, n (%)	47 (17.28%)	144 (52.94%)		93 (34.19%)	98 (36.03%)	0.396	
PLT (cells ×10^9^·L^− 1^) ( mean ± SD)	179.50 (148.25, 236.00)	179.50 (139.00, 221.00)	0.454	177.00 (138.00, 222.00)	183.00 (147.00, 223.00)	0.829	
TBiL (µmol·L^− 1^), median [IQR]	6.05 (3.70, 9.00)	3.40 (1.40, 6.40)	0.000	4.60 (2.55, 8.05)	3.20 (1.00, 6.60)	0.005	
AST (U·L^− 1^), median [IQR]	21.00 (18.75, 27.00)	22.00(18.00, 28.25)	0.803	21.00 (18.00, 27.00)	22.00 (18.00, 32.00)	0.227	
ALT (U·L^− 1^), median [IQR]	21.00 (16.00, 25.25)	21.00 (17.00, 28.00)	0.664	21.00 (17.00, 25.00)	21.00 (16.00, 29.00)	0.430	
Lactate dehydrogenase(U·L^− 1^), median [IQR]	475.00 (375.00, 581.00)	485.00 (407.00, 622.00)	0.224	471.00 (395.00, 550.00)	522.00 (402.00, 672.00)	0.013	
Serum Creatinine(µmol·L^− 1^), median [IQR]	53.00 (45.00, 58.40)	51.80 (45.30, 61.00)	0.845	51.00 (45.00, 59.00)	53.00 (45.80, 61.70)	0.131	
Uric acid(µmol·L^− 1^), median [IQR]	357.85 (323.90, 442.80)	374.60 (310.40, 442.80)	0.815	375.60 (315.25, 449.15)	363.80 (313.70, 427.80)	0.470	

ALT alanine aminotransferase, AST aspartate aminotransferase, BP blood pressure, BMI body mass index, HELLP syndrome hemolysis, elevated liver enzymes, and lower platelet count syndrome, IQR interquartile ranges, OR odds ratio, PLT platelet count, TBiltotal bilirubin

The results of the univariate and multivariable logistic regression indicated that the variables independently associated with not returning to postpartum BP follow-up visit at 6 weeks postpartum were the education level of high school or below (OR = 3.71; 95% CI = 2.01–6.85; *p* = 0.000), maximum diastolic BP during pregnancy (OR = 0.97; 95% CI = 0.94–0.99; *p* = 0.0230) and delivery gestational age (OR = 1.12; 95% CI = 1.005–1.244; *p* = 0.040). Those factors that were independently associated with not returning to postpartum BP follow-up visit at 12 weeks after childbirth included: education level of high school or below (OR = 3.20; 95% CI = 1.805–5.67; *p* = 0.000), maximum diastolic BP during pregnancy (OR = 0.95; 95% CI = 0.92–0.97; *p* = 0.000), delivery gestational age (OR = 1.13; 95% CI = 1.04–1.24; *p* = 0.006) and parity (OR = 1.63; 95% CI = 1.06–2.51; *p* = 0.026). Education level of high school or below, maximum diastolic BP during pregnancy and delivery gestational age were the common risk factors for not returning to BP follow-up visit at 6 and 12 weeks postpartum (Tables 2 and 3).

**Table 2:** Univariate analysis and Multivariable logistic regression model considering not returning to postpartum BP follow-up visit at 6 weeks postpartum

Variable	Univariate			Multivariable		
	*P*-value	OR	95% CI	*P*-value	OR	95% CI
Education level of high school or below, n (%)	0.000	3.475	1.947-6.200	0.000	3.710	2.009–6.851
Parity, n (%)	0.048	1.478	1.003–2.177			
Diastolic BP on admission(mmHg)	0.023	0.974	0.953–0.996			
Maximum diastolic BP during pregnancy(mmHg)	0.002	0.957	0.931–0.983	0.023	0.967	0.939–0.995
Severe preeclampsia as the main diagnosis at discharge, n (%)	0.001	0.396	0.225–0.697			
Combined other diagnosis at discharge, n (%)	0.088	0.430	0.163–1.135			
Delivery gestational age(weeks)	0.008	1.139	1.035–1.253	0.040	1.118	1.005–1.244
TBiL on admission (µmol·L^-1^)	0.017	1.070	1.012–1.130			

BP blood pressure, 95% CI 95% confidence intervals, OR odds ratio, TBil total bilirubin

**Table 3:** Univariate analysis and Multivariable logistic regression model considering not returning to postpartum BP follow-up visit at 12 weeks postpartum

Variable	Univariate			Multivariable		
	*P*-value	OR	95% CI	*P*-value	OR	95% CI
Education level of high school or below, n (%)	0.000	3.111	1.876–5.160	0.000	3.198	1.805–5.667
Parity, n (%)	0.000	2.085	1.419–3.063	0.026	1.630	1.060–2.507
Systolic BP on admission(mmHg)	0.011	0.982	0.967–0.996			
Diastolic BP on admission(mmHg)	0.000	0.958	0.938–0.978			
Maximum systolic BP during pregnancy(mmHg)	0.009	0.980	0.965–0.995			
Maximum diastolic BP during pregnancy(mmHg)	0.000	0.937	0.913–0.961	0.000	0.946	0.920–0.973
Gestational hypertension as the main diagnosis at discharge, n (%)	0.007	3.675	1.427–9.465			
Severe preeclampsia as the main diagnosis at discharge, n (%)	0.009	0.520	0.318–0.851			
Combined HELLP syndrome at discharge, n (%)	0.046	0.308	0.097–0.981			
Delivery gestational age(weeks)	0.001	1.146	1.058–1.241	0.006	1.134	1.037–1.239
TBiL on admission (µmol·L-1)	0.056	1.053	0.999–1.111			
Lactate dehydrogenase on admission (U·L-1)	0.007	0.998	0.997-1.000			

BP blood pressure, 95% CI 95% confidence intervals, HELLP syndrome hemolysis, elevated liver enzymes, and lower platelet count syndrome, OR odds ratio, TBil total bilirubin

**Fig. 2:**
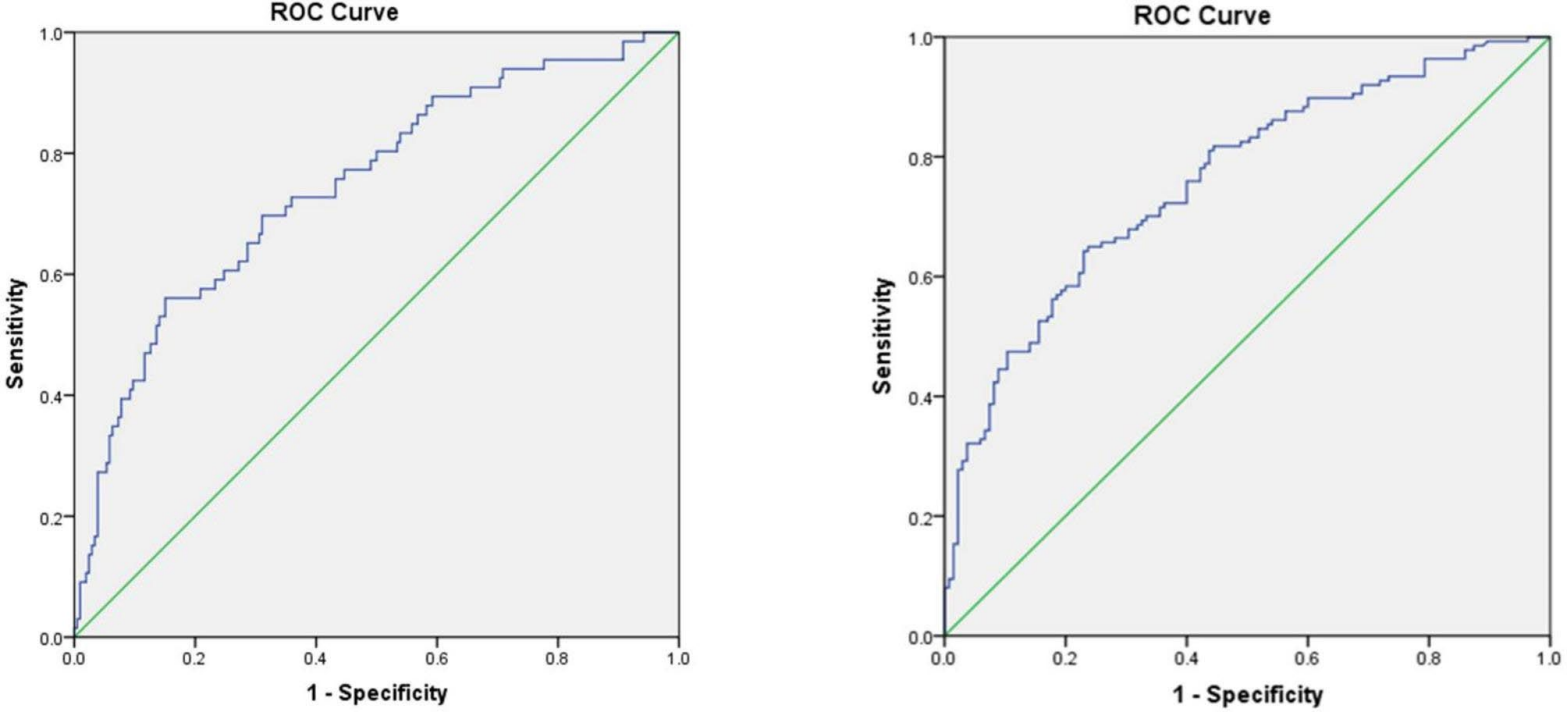
(**a**) ROC curve of the predicted probability for not returning to postpartum BP follow-up visit at 6 weeks after delivery in discharged patients with HDP. The area under the ROC curve of the predicted probability was 0.746 with sensitivity (56.1%) and specificity (85.0%), Youden index 0.410. (**b**) ROC curve of the predicted probability for not returning to postpartum BP follow-up visit at 12 weeks after delivery in discharged patients with HDP. The area under the ROC curve of the predicted probability was 0.761 with sensitivity (64.2%) and specificity (77.0%), Youden index 0.413

The specificity and sensitivity of the resulting logistic regression model were calculated for each independent risk factor of the continuous variables. This was done by constructing ROC curves and calculating the AUC to estimate the models’ ability to identify not returning to BP follow-up visit at 6 and 12 weeks postpartum (Table 4). The area under the ROC curve of the predicted probability of not returning to postpartum BP follow-up at 6 weeks after delivery was 0.746, higher than the other independent risk factors, the Youden index was 0.410, and the sensitivity and specificity were 56.1 and 85.0%, respectively. While the area under the ROC curve of the predicted probability of not returning at 12 weeks postpartum was 0.761, the Youden index was 0.413, and the sensitivity and specificity were 64.2 and 77.0% (Fig. 2). Moreover, the results showed less likelihood to return to postpartum BP visit at 6 weeks after delivery when maximum diastolic BP during pregnancy ≤ 92 mmHg or the delivery gestational age ≥ 36.36 weeks. And when the maximum diastolic BP during pregnancy was ≤ 101 mmHg, the delivery gestational age was ≥ 33.50 weeks or the parity ≥ 2, it was also less likely to return for postpartum BP follow-up visit at 12 weeks postpartum.

**Table 4:** Predicting the area under the curve and the cut-off values of the receiver operating characteristic curve of not returning to postpartum BP follow-up visit at 6 and 12 weeks after delivery in discharged patients with hypertensive disorders of pregnancy

Risk factors	AUC (95% confidence interval)	*P*-value	Cut-off point	Youden index	Sensitivity (%)	Specificity (%)
**6 weeks postpartum**						
Maximum diastolic BP during pregnancy(mmHg)	0.614 (0.528–0.699)	0.006	92	0.281	91.7%	36.4%
Delivery gestational age(weeks)	0.615 (0.539–0.692)	0.005	36.36	0.249	65.2%	59.7%
Predicted probability	0.746 (0.676–0.815)	0.000	0.34	0.410	56.1%	85.0%
**12 weeks postpartum**						
Maximum diastolic BP during pregnancy(mmHg)	0.684 (0.621–0.747)	0.000	101	0.281	73.3%	54.7%
Delivery gestational age(weeks)	0.616 (0.550–0.682)	0.001	33.50	0.180	83.2%	34.8%
Parity	0.624 (0.558–0.691)	0.000	2	0.233	74.5%	48.9%
Predicted probability	0.761 (0.704–0.817)	0.000	0.53	0.413	64.2%	77.0%

## Discussion

In this study, we identified the risk factors associated with failure to return to postpartum BP visit at 6 and 12 weeks after delivery in patients discharged with HDP, respectively. The univariate and multivariate analysis demonstrated that the education level of high school or below, maximum diastolic BP during pregnancy and delivery gestational age were identified as the significant co-variables for not returning to BP follow-up visit at 6 and 12 weeks postpartum in postpartum patients with HDP within 3 months after the discharge from the hospital.

The majority of cases of postpartum strokes and heart failure, which are often complications of hypertensive disease, have been reported to occur within 10–11 days after discharge postpartum [23], and more than 60% of deaths due to gestational hypertensive disease occur during the first 6 weeks postpartum [9]. The recommendations for monitoring hypertension in the postpartum period have started to emerge over the past decades, emphasizing the importance of postpartum follow-up [1–2, 15, 17, 24–26]. Therefore, short-interval visits to review BP logs and assess for signs or symptoms of a severe disease after delivery, so that providers can identify and address the disease before it occurs, are essential and practical.

Until now, various studies have identified predictors of 6–week postpartum visit attendance [18–20, 27–34]. It is widely recognized that postpartum visit attendance at 6 weeks is lowest among females who are non-Hispanic Black, of ethnic-minority groups, younger, multiparous, unmarried, low-income, have inadequate prenatal care use, publicly insured or uninsured, and vaginal delivery [18–20, 27–34]. However, no 12–week postpartum BP follow-up visit predictors have been reported to date. The present study adds to the sparse literature on this topic and reveals new factors affecting the return of postpartum BP follow-up visit. Lower maximum diastolic BP during pregnancy and delivery at a later median gestational age were first identified in our study as common independent risk factors at 6– and 12–week postpartum BP follow-up. Females with lower maximum diastolic BP during pregnancy and later gestational age at delivery indicate a lower severity of their HDP. It intuitively makes sense that women with less severe HDP tend not to pay enough attention to the BP follow-up visit after discharge. The results of the ROC curve analysis showed that the cut-off point of maximum diastolic BP during pregnancy at the 12–week postpartum follow-up time point was higher than that at 6–week time point, and the cut-off point of the delivery gestational age at the 12–week postpartum follow-up time point was lower than the cut-off point at 6–week time point. This suggests that even though the disease is more severe, patients with HDP place less importance on postpartum BP follow-up visit as time progresses, prompting the necessity to identify women who are less likely to return for postpartum BP visit as early as possible, so that better target interventions to improve follow up may be attempted.

The main strengths of the present study are as follows. First, this is the first prospective cohort study to report on the predictors of non-return of postpartum BP follow-up visit at each follow-up time point in postpartum discharged HDP patients and the significant data obtained through regression analysis. Second, the present results are useful for clinical practice. These results are useful to quickly identify patients who may be less likely to return for postpartum BP follow-up visit at each follow-up time point; thus, more targeted discharge education may be attempted for these patients to improve their adherence to postpartum BP follow-up.

There are, however, a few limitations to this study: (1) Our study analyzed risk factors associated with not returning to postpartum BP visit at 6 and 12 weeks after delivery in discharged patients with HDP, but it is possible that not all impact factors were included, such as data on prenatal care utilization, which was unavailable and had been identified as predictors of 6-week postpartum BP follow-up [18–20, 27–36]. (2) There was a population selection bias in our study. It is possible that those who had telephone follow up were also more likely to follow the targeted discharge education, and the region of this study was an eastern and urban area of China where people were more willing to pay more for health expenditures [37], which may also be a potential reason for the high follow-up rate in this study’s patient population. (3) This is a single-center clinical study, the demographic diversity of our cohort was narrow and these findings have limited generalizability to populations that differ demographically or geographically. (4) In this study, follow-up was conducted mainly by telephone, with limited follow-up time and patient cooperation. Therefore, due to the small sample size used, it was not possible to observe the occurrence of cardiovascular events and readmission of patients after discharge.

However, the above does not significantly affect the main results and conclusions of this study, in the future better follow-up methods will be used, such as telehealth technology, to carry out multicenter clinical cohort studies with more impact factors included to analyze and evaluate the risk factors of the adherence to BP monitoring after discharge from the hospital and to assess the long-term outcomes such as hospital readmissions, maternal mortality, and future cardiovascular health in postpartum discharged HDP patients. Reported telehealth technologies for postpartum care include call-center driven BP management [38], combining home BP cuffs with text message reminders for remote postpartum BP monitoring, or providing a Genesis Touch tablet, automatic BP cuff, scale, and pulse oximeter that allow Bluetooth transmission of all home vitals synced on a daily basis to a central monitoring platform for 2–6 weeks [39–41]. Data from these pilot studies indicated that remote BP monitoring is fully feasible and acceptable to patients and providers. It results in higher quality-adjusted years, a significant reduction in postpartum readmissions, 3.7% (8/214) versus 0.5% (1/214) [42]. Moreover, the average cost of telehealth was reported to be $309 per patient, and was cost–effective to a cost of $420 per patient. Meanwhile, telehealth could reduce health care costs in the US by approximately $31 million a year. [42].

## Conclusion

In our population, there are several risk factors associated with failure to return for BP follow-up visit at 6 and 12 weeks postpartum in discharged females with HDP. Education level at or below high school, maximum diastolic BP during pregnancy and gestational age at delivery were the common risk factors for not returning for BP follow-up visit at 6 and 12 weeks postpartum. These findings not only help to quickly identify patients who may be less likely to adhere to return for postpartum BP visits, but also suggest that we have a long way to go in improving BP follow-up attendance. Given the long-term cardiovascular risk and increased mortality in females with HDP, targeted discharge education of females before discharge to increase adherence to postpartum BP follow-up and visits are fundamental.

## Electronic supplementary material

Below is the link to the electronic supplementary material.

Supplementary Material 1

## Data Availability

The data of this study is available from the corresponding authors on reasonable request.

## Abbreviations

ACOG: The American College of Obstetricians and Gynecologists
ALT: alanine aminotransferase
AST: aspartate aminotransferase
AUC: the area under the curve
BP: blood pressure
CI: confidence intervals
HDP: hypertensive disorders of pregnancy
HELLP: hemolysis elevated liver enzymes and low platelets
IQR: interquartile ranges
OR: odds ratio
PLT: platelet count
ROC: receiver operating characteristic
SD: standard deviation
TBil: total bilirubin.
