# Supplementary material for: Risk factors associated with attendance at postpartum blood pressure follow-up visit in discharged patients with hypertensive disorders of pregnancy
Source: BMC Pregnancy Childbirth. 2023 Jun 30;23:485. doi: 10.1186/s12884-023-05780-6 (PMC10311897; doi:10.1186/s12884-023-05780-6)
Supplement: Supplementary file 1 — Supplementary Material 1 [file 12884_2023_5780_MOESM1_ESM.docx]

**Supplementary Appendix**
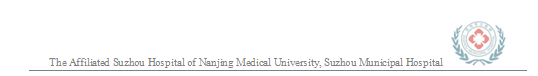


**Discharge education checklist for postpartum females with hypertensive disorders of pregnancy**

| **Name** |  | **Bed No.** |  | **Discharge date** |  |
| --- | --- | --- | --- | --- | --- |
| **Discharge diagnosis** |  | | | | |
| **Medications taken at discharge** | **Drug names and dosages** | **Precautions for medication administration** | | | |
|  |  |  | | | |
|  |  |  | | | |
|  |  |  | | | |
|  |  |  | | | |
| **Considerations for post-discharge blood pressure monitoring:**  1. **Blood pressure should be monitored at least 1 day per week for 12 weeks after delivery**, with 2-3 measurements in the morning and in the evening. Morning blood pressure measurements should be taken within 1 hour after waking up, and evening blood pressure measurements should be taken after dinner and before going to bed, after 5 minutes of rest in a sitting position, with 2 measurements at 1-minute intervals, and the average value of blood pressure monitoring should be recorded in the blood pressure record book. The record should also include: the date the blood pressure was measured and the specific time the antihypertensive medication was taken (if it was being used).  2. **The target blood pressure value is 90-140/60-90 mmHg**. If the blood pressure is not well controlled (>140/90 mmHg or <90/60 mmHg), seek prompt hospital consultation and do not use or adjust the antihypertensive drug regimen on your own.  3. **Return postpartum blood pressure** **visits are recommended at 6 and 12 weeks postpartum** to monitor and record blood pressure, and medical personnel decide whether to adjust the antihypertensive treatment regimen, including dose reduction, discontinuation, dose increase or medication change, and to determine the need for further haematological and biochemical monitoring and management.  4. **Home blood pressure measurement methods:** An upper-arm oscillometric fully automatic electronic sphygmomanometer (clinically validated sphygmomanometers are available at www.dableducational.org or www.bhsoc.org) that has been validated for standardization according to a standard protocol was selected for home blood pressure monitoring, and an appropriately sized cuff was chosen according to the circumference of the upper arm. Blood pressure should be measured in a seated position, sitting on a chair with back support, body relaxed, feet on the ground, legs uncrossed, measuring arm position in line with the level of the heart, and keeping the measurement process quiet. Loose clothing should be worn for blood pressure measurement, and the cuff should be placed over the bare arm or the arm should be removed from the clothing, with the limbs relaxed and the cuff sized appropriately, with the ventilator pointing toward the wrist, and the bottom edge of the cuff adjusted so that it is approximately 2 cm above the medial elbow joint. Tighten the cuff around the arm and secure it. The arm is placed on a table or knee with the hand slightly open and the palm facing up. The subject should rest for at least 5 minutes before the blood pressure measurement, with the cuff at the same level as the heart, and the measurement is taken by pressing the start button on the front of the sphygmomanometer. The person should remain relaxed during the measurement, do not move the arm muscles, and do not speak until the measurement is complete. At the end of the measurement the sphygmomanometer displays 3 readings: the highest value of blood pressure (systolic), the lowest value of blood pressure (diastolic) and the pulse rate, just record. The first blood pressure measurement should be completed on both upper arms, using the arm with the higher reading for subsequent blood pressure readings.  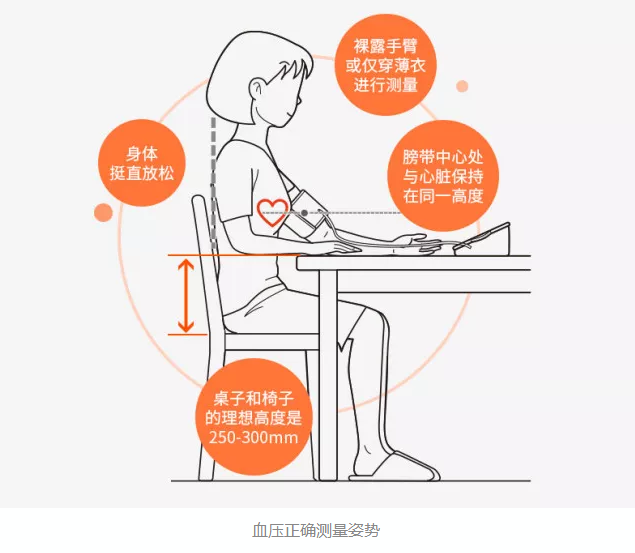  5. **If you are taking antihypertensive medication, your treatment can be adapted to accommodate breastfeeding, and that the need to take antihypertensive medication does not prevent you from breastfeeding.** Most antihypertensive medicines taken while breastfeeding only lead to very low levels in breast milk, so the amounts taken in by babies are very small and would be unlikely to have any clinical effect.  6. If you have questions about your medication or blood pressure monitoring, you are welcome to contact us at any time. The phone number of the clinical pharmacist office is 0512-62362325. You can also visit the pharmacy clinic on the second floor of the clinic for consultation at 0512-62362255, or visit the website for consultation: http://www.smh.cc/slyy/yxfw.jsp. | | | | | |
